# Surgical repair of a giant congenital right atrial aneurysm: a case report

**DOI:** 10.1186/s13019-015-0277-y

**Published:** 2015-05-14

**Authors:** Karolis Jonavicius, Arturas Lipnevicius, Rita Sudikiene, Edvardas Zurauskas, Virgilijus Lebetkevicius, Virgilijus Tarutis

**Affiliations:** 1 Faculty of Medicine Centre of Cardiac Surgery, Vilnius University, Santariskiu g. 2, Vilnius, 08661 Lithuania; 2 Department of Cardiovascular Medicine, Vilnius University, Vilnius, Lithuania; 3 Department of Pathology, Forensic Medicine and Pharmacology, Faculty of Medicine, Vilnius University, Vilnius, Lithuania

**Keywords:** Congenital right atrial aneurysm, Atrial dilatation, Heart defects

## Abstract

Giant congenital right atrial aneurysms are rare defects of the heart. Though usually asymptomatic, they can be potentially life-threatening. Major risks include heart failure, supraventricular arrhythmias, rupture of the wall of the aneurysm. This defect is usually diagnosed incidentally. It is commonly found when transthoracic echocardiography or chest X-ray is performed. In some cases computed tomography or cardiac magnetic resonance imaging is needed to establish the diagnosis. Potential therapeutic options include surgery, catheter ablation and conservative follow-up. Currently preferred method for correcting this defect is surgical excision of the aneurysm, when surgical indications are met. In this article we describe a successful aneurysmectomy performed on a 16-month old female infant, who at the time of hospitalization presented with severe heart failure and symptoms of cardiac tamponade.

## Background

Giant congenital aneurysms of the right atrium are very rare. Binder et al. reported in 2000 that from 1955 to 1998 only 60 cases of congenital enlargement of the right atrium were described in literature [1]. It can mimic all heart defects in which there is an enlargement of the right atrium such as Ebstein’s anomaly, partial anomalous pulmonary vein return, pericardial cyst or tumours of the heart [2, 3]. The diagnosis can be suspected and/or confirmed by initial transthoracic echocardiography and chest X-ray [4]. Computed tomography or cardiac magnetic resonance imaging may help distinguishing an aneurysm from cardiac tumour. Though usually discovered by accident right atrial aneurysms are potentially lethal. Binder at al. reported 5 % of deaths associated with unoperated congenital enlargement of the right atrium [1]. Major risks related giant congenital right atrial aneurysms include supraventricular arrhythmias, thrombosis, and severe atrial dilatation [1, 5–7]. In our article we present a rare case of this anomaly presenting as a tamponade, which we treated with and an complex aneurysmectomy complicated by an unexpected anatomical anomalyof the right coronary artery.

### Case report

**Fig. 1:**
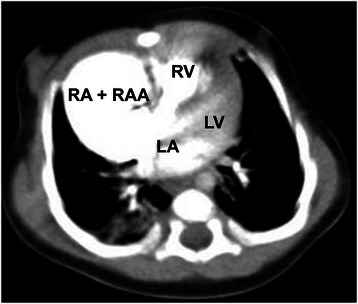
Heart CT scan. A CT of the patient’s chest at the time of diagnosis. The patient was 2 month old. The size of the aneurysm was 3.9x3.4x3.4 cm. RA + RAA indicates aneurysmatic right atrium. RV indicates right ventricle, LA – left atrium, LV – left ventricle

**Fig. 2:**
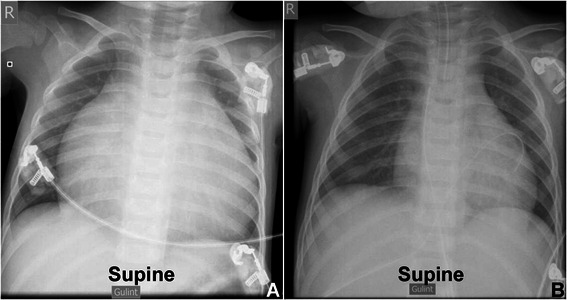
Pre-operative and Post-operative chest X-rays. **a** – Anteroposterior chest X-ray performed in supine position. Extreme cardiomegaly is visible. **b** – Anteroposterior chest X-ray performed in supine position, performed 12 h after surgery. Slight cardiomegaly is visible

A 16-month old Caucasian girl (weight 12 kg, height 95 cm, body surface area (BSA) – 0.56 m^2^) with a diagnosis of right atrial aneurysm (RAA) was transferred to our clinic. She was diagnosed with RAA at the age of two months. At that time, a computed tomography scan showed a 3.9x3.4x3.4 cm cavity directly connected to the right atrium (RA) (Fig. 1). Parents declined surgical treatment. At the time of hospitalisation she presented with “progressive” heart failure, dyspnoea and pallor. Arterial blood saturation was 98 % on room air. Initial ECG showed ectopic atrial tachycardia, which later became atrial fibrillation. Pulse rate was 110 – 160 beats per minute, arterial blood pressure was 96/68 mmHg. The lower margin of the liver was palpable at 4.5 cm below the right costal arch. Pre-operative chest X-ray showed severe cardiomegaly (Fig. 2a). Transthoracic echocardiography (TTE) revealed a large dilated RAA (7.3x5.3 cm) that was compressing both ventricles and interfering with left ventricular filling and ejection fraction (left ventricle (LV) ejection fraction (EF) was 30 %). The next day her condition started to worsen. She refused to eat. Her haemodynamics became unstable, peripheral pulses were weak and barely palpable. And her peripheral circulation was poor. Her pulse was 200 beats per minute and arterial blood pressure dropped to 60/40 mmHg. Her neck veins were congested and pulsatile. As these symptoms were consistent with cardiac tamponade a decision to perform an urgent aneurysmectomy was made.

### Surgical technique

**Fig. 3:**
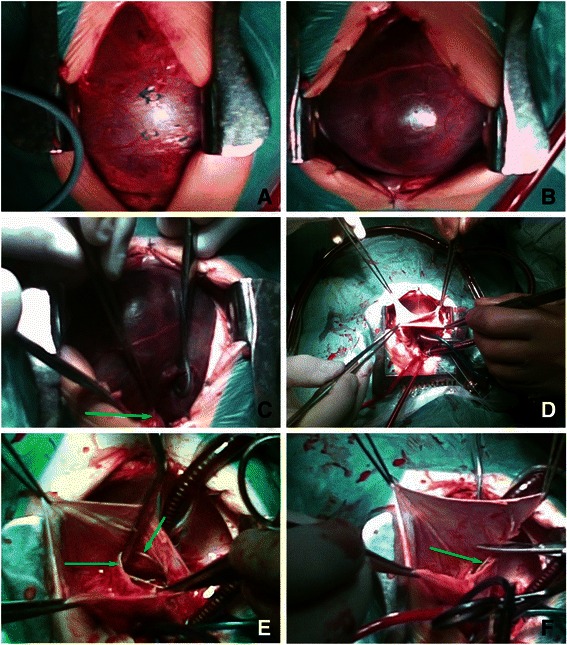
Intra-operative photographs. **a** – A view after the median sternotomy. The pericardium is distended. **b** – The pericardium was opened. The right atrial aneurysm occupies almost all pericardial space. **c** – Aortic cannula is being placed (green arrow points to the aorta). **d** – The heart was decompressed as bi-caval cardiopulmonary bypass was instituted. The aneurysm is being opened by a longitudinal incision. **e** – The aneurysm is opened and inspected. The green arrows show the protruding right coronary artery. **f** – The wall of the aneurysm is being excised above the right coronary artery (indicated by the green arrow)

**Fig. 4:**
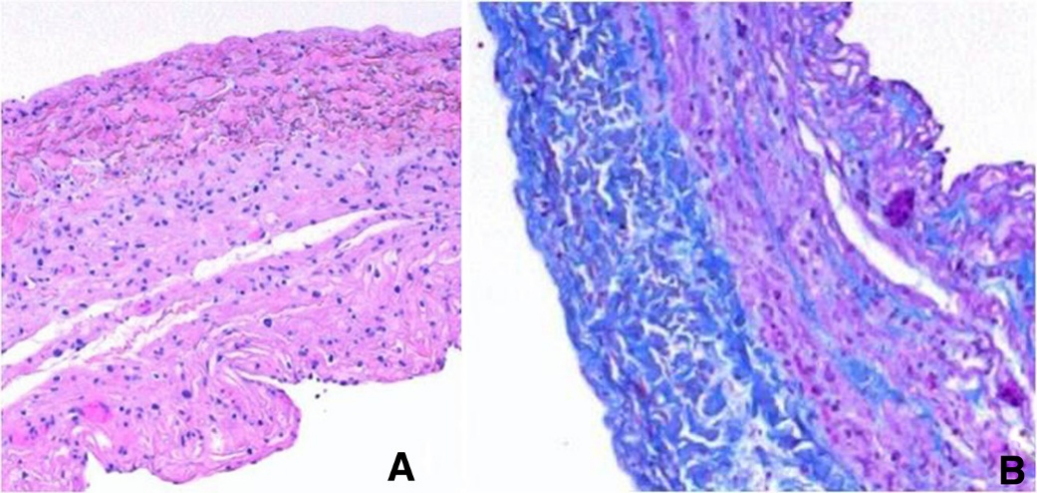
Histology of the wall of the aneurysm. **a** – In this specimen a thin wall of the resected aneurysm is visible. Almost no myofibrils are visible. An infiltration of inflammatory cells is visible. (Hematoxylin-eosin stain, original magnification x 200). **b** – Hypertrophied myofibrils with giant irregular nuclei and severe fibrosis are visible. Epicardial surface is covered by fibrin and erythrocytes (Masson trichrome stain, original magnification x 200)

The heart was reached through a median sternotomy. A huge RAA obstructed the whole operating field (Fig. 3a). The wall of the RAA was very thin and translucent and the risk of aneurysmal wall rupture was considered high (Fig. 3b). In order to decompress the heart and to safely open the aneurysm for inspection a mild hypothermic bi-caval cardiopulmonary bypass (CPB) was initiated (Fig. 3c and d). After the heart was decompressed and stopped the aneurysmal sac was opened by a longitudinal incision. Intra-operative findings were a right coronary artery (RCA) protruding onto the aneurysmal sac, normal atrial myocardium present below the crista terminalis and in the right atrial appendage and a patent foramen ovale (PFO) (Fig. 3e). The aneurysmal sac was resected (6x2.5 cm) while avoiding injuring the RCA (Fig. 3f). PFO was closed and the RA was sutured. Weaning from CPB was uneventful. Histological evaluation of the RAA sac revealed that the tissue was fibrotic, with mild lymphocytic infiltration, covered by fibrin and erythrocytes on epicardial surface. Only in small areas hypertrophic myocardial fibers were visible (Fig. 4a and b).

### Outcome and follow-up

The patient was extubated 18 h after the surgery. There were signs of right ventricle diastolic dysfunction, but they resolved in the 4th post-operative day. Post-operative chest X-ray showed a mild enlargement of the heart as seen in Fig 2b. On the 5th post-operative A TTE was performed. It showed that the RA was now only slightly enlarged (2.8x2.7 cm) and LV EF was 56 %. On the 8th post-operative day she was discharged in good general condition. The last follow-up visit at our centre took place when the patient was 6 years old. Her weight was 23 kg, height – 122 cm, BSA – 0.88 m^2^. Her growth and development were normal. TTE showed a good correction of the defect. RA size was 3.3x2.8 cm, LA size was 3.4x3 cm. LV ESD was 2.2 cm, LV EDD was 3.7 cm, LV EF was 70 %.

## Discussion

First described by Bailey in 1955, and first excised by Morrow and Behrendt in 1968 congenital right atrium aneurysm is a rare anomaly of unknown origin [8–11]. Several authors reported histological evaluation of resected aneurysmal tissue. The tissue can be normal or pathological [12]. The variety of histological changes include fibrosis, focal lymphocytic infiltration, focular myxoid changes [9, 10, 12, 13]. Forbes et al. state that histological evaluation of resected aneurysmal tissue does provide information about the cause of the anomaly [14]. In our case, the patient presented not only with arrhythmia, but also with cardiac tamponade, accompanied by marked histological pathology.

RAA can be asymptomatic or it can present with a variety of symptoms including supraventricular arrhythmias, formation of intraatrial thrombus and severe atrial dilatation. The most common one is supraventricular arrhythmias such as atrial fibrillation [1, 2, 5–7, 15]. It is not known why arrhythmias’ occur in such patients. As stated by Joshi and Pohlner it is believed that the cause of the arrhythmias’ is atrial dilatation and structural disorientation of the myocardial fibers and conduction system. This can be explained by the fact that when the RAA is removed normal sinus rhythm usually returns [15]. Forbes et al. reported a case in which the patient presented with severe and refractory arrhythmia despite of normal atrial tissue found during histological exam [14]. After the surgery our patient regained normal sinus rhythm.

There is no unified opinion on how to treat patients with RAA. There are two main approaches: conservative and surgical. Conservative approach is suggested to patients, who are asymptomatic and who are diagnosed with mild to moderate atrial dilatation. As suggested by Harder et al. those patients should receive low-dose aspirin thromboprophylaxis and they should be followed up regularly. They also emphasize that patients with severe dilatation of the atrium and symptomatic patients should be treated surgically [12]. The main indications for surgery are: atrial arrhythmia, intraatrial thrombus formation, major atrial dilatation, and compression of other heart chambers [1, 6, 7, 14]. Binder et al. state that surgical resection of right atrial aneurysm has a low mortality risk [1]. It is believed that early correction prevents further dilatation of the RA and complications associated with the presence of the RAA (such as heart failure, supraventricular arrhythmias, thrombus formation, and pulmonary or paradox embolism) [1–5, 10, 11, 15, 16]. As there are no studies that compare the two approaches we believe that each patient should be approached individually. In our case the patient was symptomatic and in critical condition. In this situation a decision to perform surgery was made as recommended by literature.

Majority of reports about this defect favours using CPB during aneurysmectomy, but Joshi and Pohlner suggest that it is safe to perform off-pump aneurysmectomy using delicate vascular clamps [2–4, 9, 12, 15, 16]. In our case we had to use CPB, because of three reasons. Firstly, the aneurysm was distended, its wall was paper-thin and risk of rupture was considered high. Secondly, open heart procedure was needed in order to close the PFO. Lastly, an unexpected finding of the protruding RCA also favoured an open heart procedure.

## Conclusions

Congenital right atrial aneurysm is a rare defect. It can be asymptomatic or present with various symptoms, supraventricular arrhythmias, intra-cardiac thrombus formation and severe atrial dilatation. Mild to moderate size asymptomatic aneurysms can be treated conservatively with low-dose aspirin thromboprophylaxis and regular follow-up. Large and symptomatic aneurysms should be excised during surgery. If there are no other heart defects present, it is possible to remove an aneurysm of the right atrium without use of cardiopulmonary bypass. Though, open heart aneurysmectomy using cardiopulmonary bypass provides the means of safe investigation of the aneurysmal sac and repair of other heart defects that may be present.

## Consent

Written informed consent was obtained from the patients legal guardians for publication of this case report and any accompanying images.

## Abbreviations

BSA: Body surface area
RAA: Right atrial aneurysm
RA: Right atrium
TTE: Transthoracic echocardiography
LV: Left ventricle
LV ESD: Left ventricle end-systolic diameter
LV EDD: Left ventricle end-diastolic diameter
LV EF: Left ventricle ejection fraction
CPB: Cardiopulmonary bypass
RCA: Right coronary artery
PFO: Patent foramen ovale
LA: Left atrium
